# Avian influenza A (H9N2) virus infections among poultry workers, swine workers, and the general population in Beijing, China, 2013‐2016: A serological cohort study

**DOI:** 10.1111/irv.12641

**Published:** 2019-03-18

**Authors:** Chunna Ma, Shujuan Cui, Ying Sun, Jiachen Zhao, Daitao Zhang, Li Zhang, Yi Zhang, Yang Pan, Shuangsheng Wu, Wei Duan, Man Zhang, Peng Yang, Quanyi Wang

**Affiliations:** ^1^ Institute for Infectious Disease and Endemic Disease Control Beijing Municipal Center for Disease Prevention and Control Beijing China; ^2^ Institute for Infectious Disease and Endemic Disease Control Beijing Research Center for Preventive Medicine Beijing China; ^3^ School of Public Health Capital Medical University Beijing China

**Keywords:** general population, H9N2, infection, poultry workers, swine workers

## Abstract

**Background:**

Few studies have reported on the seroprevalence of antibodies against avian influenza A (H9N2) virus and the incidence of these infections in the northern China and among swine workers.

**Methods:**

We conducted a serological cohort study among people working with poultry or swine or the general population in Beijing, China. It comprised four cross‐sectional serological surveys in November 2013, April 2014, April 2015, and April 2016. Blood samples collected from the participants were tested for anti‐H9N2 antibodies using a hemagglutination‐inhibition (HI) assay. Multivariable Poisson regression model was then used to compare the person‐month incidence rates for H9N2 viral infections among the three groups, assessed by incidence rate ratio (IRR).

**Results:**

In the four cross‐sectional surveys, the highest seroprevalence of anti‐H9N2 antibodies (HI titer ≥ 80) was recorded in the poultry workers (2.77%, 19/685) in April 2016, while the lowest was recorded in the general population (0.09%, 1/1135) in April 2015. The highest incidence density rate for H9N2 infections across the whole study period was recorded among the poultry workers (3.75/1000 person‐months), followed by the swine workers (1.94/1000 person‐months) and the general population (1.78/1000 person‐months). Multivariable analysis showed that the poultry workers were at higher risk (IRR: 2.42, 95% CI: 1.07‐5.48; *P* = 0.034) of contracting H9N2 virus than the general population.

**Conclusions:**

Although the seroprevalence of H9N2 antibodies was low in Beijing, the poultry workers were at higher risk of contracting H9N2 viral infections than the general population. Closer monitoring and strengthened protection measures for poultry workers are warranted.

## 1 INTRODUCTION

The first recorded avian influenza A (H9N2) virus infections occurred in 1966 among turkeys in the United States.^1^ Since the 1990s, this virus has readily circulated among domestic poultry in several Asian countries and is now considered to have a near global distribution in poultry with sporadic or regional outbreaks.^2^ H9N2 virus infections are continuously found in poultry (chickens, ducks, quail, partridges, chukar, pheasants, guinea fowl, and pigeons),^3^ wild birds, domestic mammals (dogs, cats),^4^ and occasionally humans.^5^, ^6^ The majority of viruses that have been sequenced belong to the A/quail/Hong Kong/G1/97 (G1), A/chicken/Beijing/1/94 (Y280/G9), or Eurasian clades.^7^ One study on avian influenza A (H9N2) virus evolution showed that an emerging genotype (G57) had increased the infectivity of this virus in chickens via its altered antigenicity and improved adaptability in these birds.^8^ H9N2 virus is widely prevalent in poultry in Asia, including China.^9^ In fact, H9N2 virus has become the most prevalent avian influenza virus (AIV) in Chinese poultry.^10^ The hemagglutination (HA) gene sequences from the influenza virus resource database at the US National Center of Biotechnology Information (NCBI) indicate that more than 90% of the globally isolated H9N2 viruses come from Asia, of which 78% come from China.^11^

The first known human infection with H9N2 virus was reported in 1998 in Guangdong province, China^11^, ^12^, ^13^. Because the clinical symptoms of most of the H9N2 human cases are mild, it is difficult to identify them through regular surveillance systems.^5^ A systematic review and meta‐analysis indicated that the seroprevalence of antibodies to H9N2 virus ranged from 0.6% to 42.6% (median, 4.9%).^2^ Notably, in mainland China, over 75% of poultry H9N2 viruses possess a Q226L mutation at residue 226 in the HA receptor‐binding site (RBS).^14^ Unlike some AIV subtypes that preferentially bind to *α*2,3‐linked sialic acids (Sia*α*2,3Gal), the Q226L substitution in the H9N2 HA gene enhances the binding of HA to the terminal *α*2,6‐linked sialic acids (Sia*α*2,6Gal) that are predominantly expressed the upper respiratory tracts of humans and swine,^15^, ^16^ whereas most human and swine influenza viruses tend to prefer to bind to receptors containing Sia*α*2,6Gal. Therefore, the switch from the Sia*α*2,3 Gal RBS to the Sia*α*2,6Gal RBS is an important step for AIV adaptation to mammals.

Eight migratory routes for wild birds exist in the world, and China is located in three of them: the East Asia–Australia Flyway, Central Asia Flyway, and the West Asia‐East Africa flyway. Lakes and related wetlands along the flyways (eg, Qinghai Lake Nature Reserve,^17^ Dongting Lake Nature Reserve,^18^ and Poyang Lake Nature Reserve^19^) are very important staging, overwintering and breeding sites for migratory birds. Each migration season, tens of millions of wild birds (>10 million birds for Dongting Lake) congregate at the lakes, sharing a common habitat with local birds, including domestic ducks.^20^ The mixed environment provides an opportunity for AIV transmission among wild birds, local birds, and domestic fowl,^20^ thereby increasing the risk of virus reassortment,^21^ making China an epicenter for AIV.^10^ The H9N2 virus is now stably established in chicken flocks and is endemic across the vast majority of China,^9^, ^22^ occurring in live poultry markets, backyard flocks, and other environments,^23^, ^24^ making its transmission from poultry to humans more likely and increasing the chance of viral mutation and gene reassortment.^25^, ^26^ In addition, improved influenza surveillance in humans also contributes to the observed increase in human infections with H9N2. It should also be noted that some of the H9N2 viruses display the human influenza virus‐like receptor specificity described above, and H9 subtype AIVs are therefore considered to be one of the most likely candidates for a new influenza pandemic in humans.^27^ Concurrently, avian H9N2 has also donated its six internal genes to H5N1,^28^ H7N9,^8^, ^10^, ^29^ and H10N8^10^, ^30^ human‐infecting viruses, resulting in their ability to transfer naturally to humans and also enhancing the likelihood of other AIVs becoming pandemic strains.

To date, most research on H9N2 virus antibody levels in China only cover the southern Chinese provinces and have only been conducted in poultry workers. Studies on the infection in the northern Chinese provinces and among swine workers are limited in number. Serological studies can identify asymptomatic or mild infections in the population that are easily missed by surveillance systems. Therefore, to evaluate the level of H9N2 virus infection among the at‐risk population in northern China, we conducted a serological cohort study in poultry workers, swine workers, and the general population in Beijing, China, where live poultry markets were banned after 2005.

## 2 METHODS

### 2.1 Study design

This serological cohort study was implemented among poultry workers, swine workers, and the general population in Beijing, China, and included four serological surveys conducted in November 2013, April 2014, April 2015, and April 2016. In the first survey (November 2013), the participants were recruited and invited to complete a questionnaire and provide serum samples for antibody detection of AIVs. In the second survey (April 2014), the people who participated in the first survey in 2013 were followed up to complete another questionnaire and provide serum samples. Because some participants were lost to follow‐up, some other people who had similar occupational characteristics and the same workplace as the dropped‐out participants were enrolled into the second survey to compensate for the reduced sample size. For the third survey (April 2015), the people who participated in the second survey in April 2014 were followed up to complete the third questionnaire and provide serum samples. Similarly, some other people were recruited to participate in the third survey to compensate for the lost study participants. In the last survey (April 2016), the people who participated in the third survey in April 2015 were followed up to complete a questionnaire and provide serum samples. No additional people were invited to participate in the final survey.

People who participated in both surveys of November 2013 and April 2014 are referred to here as the 2013‐2014 cohort, and those who participated in both surveys of April 2014 and April 2015 are referred to as the 2014‐2015 cohort; likewise, people who participated in both surveys of April 2015 and April 2016 are referred to as the 2015‐2016 cohort.

### 2.2 Participant selection

Multistage cluster sampling was used to recruit poultry‐related workers and swine‐related workers, and a multistage stratified random sampling technique was used to enroll the general population.

First, to ensure sufficient numbers of subjects could be enrolled, five agriculture‐related districts in Beijing, China, were selected by convenience sampling. Every district covered six types of sites and seven kinds of people (ie, large‐scale poultry‐breeding enterprises, large‐scale poultry abattoirs, private poultry farms or backyard poultry raising sites, large‐scale pig‐breeding enterprises, large‐scale pig‐abattoirs, private pig farms, or backyard pig raising sites). Second, (a) for workers in large‐scale poultry/swine‐breeding enterprises, two poultry and two swine commercial breeding enterprises were selected in each of the five districts, and all poultry and swine workers from the selected commercial breeding enterprises were invited to participate in the study; (b) for workers in large‐scale poultry/swine‐abattoirs, two poultry and two swine commercial abattoirs were selected in each of the five districts, and all poultry and swine workers from the selected commercial abattoirs were invited to participate in the study; (c) for farmers in private poultry/swine farms or backyard poultry/pig raising sites, two towns with poultry industries and two towns with swine industries were selected in each of the five districts, and all the workers from the private poultry/swine farms or backyard poultry/pig raising sites in the selected towns were invited to participate in the study; (d) the general population (defined here as individuals not engaged in poultry‐related and swine‐related work, or were not breeding poultry/swine in their backyards) were enrolled from the same districts as the poultry and swine‐related workers. The participants were selected using the random number methodology shown below:

- One town and one street in each of the five districts;
- Two villages from each town and two communities from each street chosen;
- Sixty individuals aged above 18 years in each village or community.

Participants in some other serological studies on H9N2 AIV included live poultry market workers. Unfortunately, our study did not involve live poultry market workers, because live poultry markets were banned after 2005 in Beijing.

### 2.3 Data collection and serum collection

Trained staff employed a standardized questionnaire to collect the epidemiological and clinical data from the study participants (eg, demographic characteristics and underlying medical conditions). Chronic diseases in the participants were defined as any one of the following: asthma, tuberculosis, pulmonary fibrosis, chronic tracheitis or bronchitis, emphysema, chronic obstructive pulmonary disease, diabetes, anemia, oncological diseases, immune system diseases, cardiovascular and cerebrovascular diseases, renal diseases, hepatopathy, and neurological diseases.

Nurses collected a 5 mL blood sample from every participant in each serological survey and transported these samples to the laboratory of the corresponding district's Center for Disease Prevention and Control (CDC). Serum from each blood sample was stored at −80°C and transported to the Beijing CDC for antibody testing against H9N2 virus.

### 2.4 Laboratory testing

Because this study included four large‐scale seroepidemiological surveys, to ensure its feasibility and validity, a hemagglutination‐inhibition (HI) assay was employed with higher efficiency than a microneutralization (MN) assay in lieu of MN. Serum samples obtained from the study participants were assayed for antibodies against H9N2 virus using a HI assay method described in the World Health Organization Manual.^31^ All the serum samples were pre‐treated with receptor destroying enzyme to remove non‐specific inhibitors and absorbed onto turkey erythrocytes to remove non‐specific agglutinins. Each pre‐treated serum sample was diluted 1:10 dilution to test for specific antibodies against H9N2 virus antigens using a 1% volume of turkey erythrocytes. An H9N2 virus strain isolated by our laboratory (A/environment/Beijing/w001/2013 H9N2), representative of the circulating viruses at the time of the study, was used as the H9N2 virus antigens for the HI assay. The complete HA gene sequence for this H9N2 virus was submitted to the Global Initiative on Sharing All Influenza Data Repository (GISAID, EPI1353255). The sequences of HA gene of the H9N2 virus used in our study and the viruses circulating in Beijing in recent years that could be detected in all seasons belong to the same clade (clade 4.2.5).^32^ HI titers of 80 and 160 were considered to be the cutoff titers for determining seropositivity in the four independent surveys.^33^, ^34^ Antibody seroconversion against the H9N2 virus involving a 4‐fold or greater increase between the paired serum samples with titers of ≥40 for the second specimen^35^ was considered to be a new infections in the cohort study. Positive control (HI titer, 640) and negative control sera were included in each run.

### 2.5 Data analysis

Data were analyzed using spss V.20.0 (IBM Corporation, New York, NY, USA). Participants who had missing demographic characteristics data or underlying medical conditions were retained in the study, but those with missing HI titer data were excluded. In each cross‐sectional survey, the seropositivity determinations depended on whether the HI titer of a single serum sample from a participant in this survey was equal or greater than the cutoff titer, regardless of the results for the sera collected in the other surveys, even when this participant took part in the other surveys. In each cohort, the seroconversion determination depended on the comparison between the paired serum samples collected at the beginning and end for each cohort, irrespective of the serological results of the other cohorts (Appendix S1). Seroprevalence rates were used to estimate the previous infection status in the four cross‐sectional serological surveys. Seroconversion was considered to be the serological evidence of a new infection with H9N2 virus during the follow‐up period for each cohort (eg, the 2013‐2014, 2014‐2015, and 2015‐2016 cohorts). The person‐time incidence rates (incidence density rates) were calculated as follows: the total number of new infections in the three cohorts (2013‐2014, 2014‐2015, and 2015‐2016 cohorts) divided by the total number of person‐months of follow‐up. Percentages were calculated for the categorical variables. Proportions were compared using Pearson's chi‐square test, and Fisher's exact test. Multivariable Poisson regression models were performed to compare the person‐month incidence rates for H9N2 infections among the three groups of people, as assessed by the incidence rate ratio (IRR). All tests were two‐sided, and statistical significance was defined as *P* < 0.05.

### 2.6 Ethical statement

The Institutional Review Board and the Human Research Ethics Committee of the Beijing CDC provided ethical approval for this study. Informed consent was obtained from all the participants before interview and blood collection.

## 3 RESULTS

### 3.1 Characteristics of the participants

**Figure 1:**
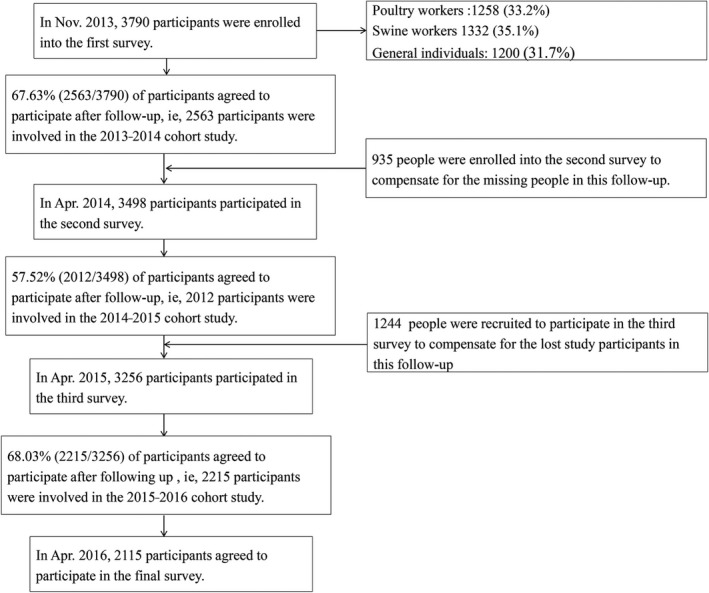
Subject enrolment flowchart for the cross‐sectional and cohort study aimed at estimating the infection risk of H9N2 virus among poultry workers, swine workers and the general population in Beijing, China

In November 2013, a total of 3790 participants were enrolled into the first survey. In April 2014, 3498 participants, consisting of 2563 people who participated in the first survey and 935 new recruits, participated in the second survey. In April 2015, 3256 participants participated in the third survey, which included 2012 people from the second survey and 1244 new recruits. In April 2016, 2215 recruits agreed to participate in the fourth survey after follow‐up (Figure 1).

Among the four independent surveys, the distribution of participants differed significantly by participant category (*P* < 0.001), age group (*P* = 0.004), and the presence of at least one chronic disease (*P* < 0.001). These differences also existed in the three cohorts in the years 2013‐2014, 2014‐2015, and 2015‐2016 (Table 1).

**Table 1:** Characteristics of the participants in the four cross‐sectional surveys in 2013, 2014, 2015, and 2016, and the respective cohorts during 2013‐2014, 2014‐2015, and 2015‐2016

Characteristics	Cross‐sectional surveys				*P* value	Cohorts (y)			*P* value
	Nov 2013	Apr 2014	Apr 2015	Apr 2016		2013‐2014 (Nov 2013‐Apr 2014)	2014‐2015 (Apr 2014‐Apr 2015)	2015‐2016 (Apr 2015‐Apr 2016)	
Participant category									
Poultry workers	1258 (33.2)	1056 (30.2)	1123 (34.5)	685 (30.9)	<0.001	791 (30.9)	512 (25.4)	685 (30.9)	<0.001
Swine workers	1332 (35.1)	1254 (35.8)	998 (30.7)	610 (27.5)		862 (33.6)	568 (28.2)	610 (27.5)	
General population	1200 (31.7)	1188 (34.0)	1135 (34.8)	920 (41.5)		910 (35.5)	932 (46.3)	920 (41.5)	
Gender									
Male	1933 (51.0)^a^	1686 (48.3)^a^	1595 (49.0)^a^	1062 (48.1)^a^	0.062	1224 (47.8)^a^	941 (46.8)^a^	1062 (48.1)^a^	0.671
Female	1855 (49.0)^a^	1807 (51.7)^a^	1658 (51.0)^a^	1145 (51.9)^a^		1338 (52.2)^a^	1068 (53.2)^a^	1145 (51.9)^a^	
Age group									
≤39	1244 (32.9)^a^	1117 (32.0)^a^	1027 (31.6)^a^	556 (25.2)^a^	<0.001	790 (30.9)^a^	547 (27.2)	556 (25.2)^a^	<0.001
40‐59	1925 (50.9)^a^	1760 (50.4)^a^	1577 (48.6)^a^	1109 (50.3)^a^		1302 (50.9)^a^	1002 (49.8)	1109 (50.3)^a^	
≥60	616 (16.3)^a^	613 (17.6)^a^	643 (19.8)^a^	541 (24.5)^a^		467 (18.2)^a^	463 (23.0)	541 (24.5)^a^	
Presence of at least one chronic disease									
Yes	284 (7.5)	177 (5.1)	291 (8.9)	209 (9.4)	<0.001	200 (7.8)	117 (5.8)	212 (9.6)	<0.001
No	3506 (92.5)	3321 (94.9)	2965 (91.1)	2006 (90.6)		2263 (92.2)	1895 (94.2)	2003 (90.4)	
Total	3790 (100)	3498 (100)	3256 (100)	2215 (100)		2563 (100)	2012 (100)	2215 (100)	

Numbers in parentheses are proportion (%) of participants, unless otherwise indicated.

a Variables with missing data.

Among the poultry workers, swine workers, and the general population in this cohort study, the distribution of person‐months differed significantly by gender, age group, and the presence of at least one chronic disease (Table 2).

**Table 2:** Characteristics of the poultry workers, swine workers, and the general population who participated in the cohort study

Characteristics	Poultry workers	Swine workers	General population	*P* value
	person‐months (%)	person‐months (%)	person‐months (%)	
Gender				
Male	6757 (40.44)^a^	9231 (54.35)^a^	11 801 (47.72)^a^	<0.001
Female	9950 (59.56)^a^	7752 (45.65)^a^	12 927 (52.28)^a^	
Age group				
≤39	3770 (22.57)^a^	3822 (22.52)^a^	8166 (32.99)	<0.001
40‐59	9076 (54.33)^a^	10 052 (59.22)^a^	10 217 (41.28)	
≥60	3859 (23.10)^a^	3101 (18.26)^a^	6369 (25.73)	
Presence of at least one chronic disease				
Yes	1117 (6.62)	512 (3.01)	2886 (11.66)	<0.001
No	15 761 (93.38)	16 482 (96.99)	21 866 (88.34)	
Total	16 806 (100)	16 994 (100)	24 752 (100)	

a Variables have missing data.

### 3.2 Seroprevalence of anti‐H9N2 antibodies in the four cross‐sectional surveys

In the four cross‐sectional surveys, the seroprevalence of anti‐H9N2 antibodies (HI titer ≥80) ranged from 0.48% to 2.77% in the poultry workers, from 0.60% to 1.35% in the swine workers, and from 0.09% to 1.18% in the general population. A similar trend was seen for anti‐H9N2 antibody seroprevalence based on a HI titer of ≥160, which ranged from 0.08% to 1.02% in the poultry workers, from 0.20% to 0.49% in the swine workers, and from 0% to 0.59% in the general population. During the study period, the seroprevalence trends did not increase or decrease among three groups over time. The highest seroprevalence of anti‐H9N2 antibodies (HI titer ≥80) was recorded among the poultry workers in April 2016 (2.77%, 19/685), and the lowest was recorded among the poultry workers in April 2015 (0.09%, 1/1135). The highest anti‐H9N2 antibody seroprevalence (HI titer ≥160) was recorded among the poultry workers in April 2016 (1.02%, 7/685), and the lowest was recorded among the poultry workers in April 2015 (0%, 0/1135) (Table 3).

**Table 3:** Seroprevalences of antibodies (HI titer ≥80 and ≥160) to H9N2 virus in poultry workers, swine workers, and the general population in four cross‐sectional surveys in 2013, 2014, 2015, and 2016

Characteristic/Period	Nov 2014				Apr 2014				Apr 2015				Apr 2016			
	HI titer ≥80		HI titer ≥160		HI titer ≥80		HI titer ≥160		HI titer ≥80		HI titer ≥160		HI titer ≥80		HI titer ≥160	
	N. (%)	*P* value	N. (%)	*P* value	N. (%)	*P* value	N. (%)	*P* value	N. (%)	*P* value	N. (%)	*P* value	N. (%)	*P* value	N. (%)	*P* value
All participants																
Participant category	30 (0.79)		5 (0.13)		45 (1.29)		14 (0.40)		20 (0.61)		5 (0.15)		27 (1.22)		10 (0.45)	
Poultry workers	6 (0.48)	0.017	1 (0.08)	0.631^b^	17 (1.61)	0.531	3 (0.28)	0.473^b^	13 (1.16)	0.005	3 (0.27)	0.245^b^	19 (2.77)	＜0.001	7 (1.02)	0.003^b^
Swine workers	18 (1.35)		3 (0.23)		14 (1.12)		4 (0.32)		6 (0.60)		2 (0.2)		5 (0.82)		3 (0.49)	
General population	6 (0.50)		1 (0.08)		14 (1.18)		7 (0.59)		1 (0.09)		0 (0)		3 (0.33)		0 (0)	
Gender^a^																
Male	19 (0.98)	0.176	2 (0.10)	0.681^b^	25 (1.48)	0.325	7 (0.42)	0.897	7 (0.44)	0.208	1 (0.06)	0.375^b^	13 (1.22)	0.847	6 (0.56)	0.327^b^
Female	11 (0.59)		3 (0.16)		20 (1.11)		7 (0.39)		13 (0.78)		4 (0.24)		13 (1.14)		3 (0.26)	
Age group^a^																
≤39	6 (0.48)	0.266	1 (0.08)	0.858^b^	12 (1.07)	0.056	5 (0.45)	0.679^b^	8 (0.78)	0.687	1 (0.10)	0.513^b^	5 (0.90)	0.515	3 (0.54)	0.768^b^
40‐59	17 (0.88)		3 (0.16)		19 (1.08)		8 (0.45)		8 (0.51)		2 (0.13)		13 (1.17)		4 (0.36)	
≥60	7 (1.14)		1 (0.16)		14 (2.28)		1 (0.16)		4 (0.62)		2 (0.31)		9 (1.66)		3 (0.55)	
Presence of at least one chronic disease																
Yes	3 (1.06)	0.489^b^	1 (0.35)	0.323^b^	4 (2.26)	0.286^b^	0 (0.00)	1.000^b^	0 (0.00)	0.250^b^	0 (0.00)	1.000^b^	1 (0.48)	0.507^b^	1 (0.48)	
No	27 (0.77)		4 (0.11)		41 (1.23)		14 (0.42)		20 (0.67)		5 (0.17)		26 (1.30)		9 (0.45)	1.000^b^

a Variables have missing data.

b Using Fisher's exact test.

### 3.3 Incidence of infections with H9N2 virus in the cohorts during the years 2013‐2014, 2014‐2015, and 2015‐2016 (November 2013 to April 2016)

In the cohorts followed up during 2013‐2014, 2014‐2015, and 2015‐2016, the overall incidence density rate for H9N2 infections was 2.39 per 1000 person‐months (140/58 552). The highest incidence density rate for H9N2 infections was recorded among the poultry workers (3.75 per 1000 person‐months), followed by swine workers (1.94 per 1000 person‐months) and the general population (1.78 per 1000 person‐months). Poisson regression analysis also showed that the poultry workers were at higher risk (IRR: 2.42, 95% CI: 1.07‐5.48; *P* = 0.034) of contracting H9N2 than the general population (Table 4), but no statistically significant difference was identified between the swine workers and general population (IRR: 0.35, 95% CI: 0.09‐1.36; *P* = 0.128). There were also no statistically significant differences between the subgroups stratified by gender, age group, and chronic diseases (*P *>* *0.05; Table 4).

**Table 4:** Incidence density rates over time and infection risk of H9N2 virus in the cohort study in Beijing, China, from November 2013 to April 2016

Characteristic	2013‐2014 y			2014‐2015 y			2015‐2016 y			All of 3 y		
	Number of infections^a^/person‐months(‰)	Adjusted IRR^b^ (95% CI)	*P* value	Number of infections^a^/person‐months(‰)	Adjusted IRR^b^ (95% CI)	*P* value	Number of infections^a^/person‐months(‰)	Adjusted IRR^b^ (95% CI)	*P* value	Number of infections^a^/person‐months(‰)	Adjusted IRR^b^ (95% CI)	*P* value
Participant category												
Poultry workers	20/3065 (6.53)	1.06 (0.4‐2.84)	0.901	20/6002 (3.33)	5.00 (0.87‐28.86)	0.072	23/7739 (2.97)	3.26 (0.43‐24.68)	0.253	63/16 806 (3.75)	2.42 (1.07‐5.48)	0.034
Swine workers	13/3402 (3.82)	0.07 (0‐1.13)	0.061	11/6671 (1.65)	1.84 (0.32‐10.63)	0.495	9/6921 (1.30)	0.53 (0.02‐13.01)	0.697	33/16 994 (1.94)	0.35 (0.09‐1.36)	0.128
General population	26/3453 (7.53)	Ref		13/10 877 (1.20)	Ref		5/10 422 (0.48)	Ref		44/24 752 (1.78)	Ref	
Gender												
Male	35/4763 (7.35)	Ref		19/11 001 (1.73)	Ref		17/12 025 (1.41)	Ref		71/27 789 (2.55)	Ref	
Female	24/5153 (4.66)	0.45 (0.14‐1.42)	0.175	25/12 514 (2)	0.57 (0.07‐4.3)	0.583	18/12 962 (1.39)	0.32 (0.01‐7.79)	0.482	67/30 629 (2.19)	0.45 (0.16‐1.23)	0.120
Age group												
≤39	19/3104 (6.12)	Ref		12/6350 (1.89)	Ref		8/6304 (1.27)	Ref		39/15 758 (2.47)	Ref	
40‐59	25/5031 (4.97)	0.42 (0.12‐1.45)	0.168	18/11 741 (1.53)	1.31 (0.26‐6.62)	0.747	16/12 573 (1.27)	0.29 (0.01‐7.09)	0.447	59/29 345 (2.01)	0.52 (0.20‐1.35)	0.180
≥60	15/1769 (8.48)	0.36 (0.06‐2.04)	0.248	14/5459 (2.56)	1.61 (0.28‐9.31)	0.593	13/6101 (2.13)	1.47 (0.15‐14.12)	0.739	42/13 329 (3.15)	0.63 (0.21‐1.86)	0.339
Presence of at least one chronic disease												
Yes	54/9158 (5.9)	2.97 (0.17‐51.51)	0.454	42/22 181 (1.89)	10.44 (0.5‐217.48)	0.130	33/22 698 (1.45)	14.75 (0.6‐361.95)		129/54 037 (2.39)	2.11 (0.12‐35.82)	0.605
No	5/762 (6.56)	Ref		2/1369 (1.46)	Ref		4/2384 (1.68)	Ref	0.099	11/4515 (2.44)	Ref	

CI: confidence interval; IRR: incidence rate ratio.

a Antibody seroconversion against the H9N2 viruses was considered as infection and defined as a 4‐fold or greater increase in antibody titer by hemagglutination‐inhibition test between the paired serum samples with titers ≥ 40 for the second specimen.

b Multivariable Poisson regression model was used to compare the person‐month incidence rates for H9N2 infections among various populations by adjusting gender, age group, and presence of at least one chronic disease.

## 4 DISCUSSION

Our results have shown that the risk of infection with H9N2 virus among poultry workers, swine workers, and the general population in Beijing, China, has remained low since 2013, as indicated by the antibody seroprevalence in the four cross‐sectional surveys as well as the infection incidence rates in the cohorts during 2013‐2014, 2014‐2015, and 2015‐2016. Our study has also revealed that the poultry workers were at higher risk than the general population of contracting H9N2 virus.

We also found that the seroprevalence of anti‐H9N2 antibodies in the poultry workers ranged from 0.48% to 2.77% in accordance with a HI cutoff titer of 80, or from 0.08% to 1.02% in accordance with a HI cutoff titer of 160. The seroprevalences in this study were lower than those of a meta‐analysis study (4.9%), in which the seroprevalence ranged from 0.6% to 42.6% among the avian‐exposed populations, as reported in the studies published during 1997‐2013, which involved Asia, the Middle East, Africa, and parts of North America^2^ (based on HI assays for all the studies; the HI cutoff titers varied in the studies, ranging from 20 to 160). A cross‐sectional survey of farm poultry workers in Pakistan reported that the seroprevalence was 47.8% for H9^36^ (HI assay; HI cutoff titer of 160). Similarly, in our cohort study, the incidence rate of H9N2 virus infection among poultry workers in Beijing from November 2013 to April 2016 was much lower than that observed in some other countries. A prospective, controlled seroepidemiological study conducted in Egypt found that seroprevalence of A (H9N2) among people exposed to poultry was between 5.6% and 7.5%^37^ (HI assay; HI cutoff titer, 80). A longitudinal cohort study conducted on Vietnamese farming households reported a 9% value for subclinical seroconversions to A/H9 between 2013‐2015^38^ (HI assay; HI cutoff titer, 40).

The seroprevalences of H9N2 infections in southern China (3.42% for A/Guangzhou/333/99(G9); 1.37% for A/quail/Hong Kong/G1/97(G1)) were significantly higher than those in northern China (2.34% for A/Guangzhou/333/99 [G9]; 0.81% for A/quail/Hong Kong/G1/97 [G1]).^11^ In contrast, H9N2 seroprevalence in Beijing (northern China) was lower than that in the southern Chinese cities, but approached that in the northern Chinese province of Shandong (0.8%) (HI assay; HI cutoff titer, 80).^11^, ^39^ Three possibilities exist for the lower seroprevalence of H9N2 in Beijing than in the southern Chinese areas. First, the circulation intensity of H9N2 in the northern provinces was lower than in the southern provinces. Southern China has a higher density of live poultry sales and poultry farming than northern China, making it a reservoir for AIVs.^11^ Second, our study did not involve workers from live poultry markets, because these markets were banned in Beijing after 2005, but the participants in some of the other serological studies on H9N2 AIV included live poultry market workers. Furthermore, a cross‐sectional, seroepidemiological study conducted in Guangdong Province showed that the seroprevalence of anti‐H9N2 antibodies in the poultry market workers was much higher than in the poultry farm workers and veterinary staff,^40^ a finding consistent with the results of another study.^41^ Third, people in the southern provinces prefer to eat fresh rather than frozen poultry, and this increases their exposure to the virus. However, it should be noted that differences in laboratory methods and cutoff titers may influence the accuracy of the comparisons between the studies.

The incidence density rate for H9N2 viral infections (from 2013 to 2015, 3.08 per 1000 person‐months) among all the study participants was found to be higher than those for H7N9 (0.4 per 1000 person‐months) and H5N1 (1.3 per 1000 person‐months) infections observed in the same period with the same population^42^ (HI assay; HI cutoff titer, 80). Another study conducted in Guangdong Province, China, also revealed that the seroprevalence of anti‐H9N2 antibodies (6.79%) was higher than for H7N9 (3.95%), H5N1 (1.36%), and even avian‐like canine H3N2 (1.85%)^40^ (HI assay; HI cutoff titer, 40). A prospective, controlled, seroepidemiological Egyptian study also reported that the seroprevalence of H9N2 among exposed humans was 5.6%‐7.5% higher than for anti‐A (H5N1) antibodies (2%) (MN assay).^37^ A Vietnamese seroprevalence study conducted in 2001 showed that the seroprevalence rates for H5 and H9 antibodies were 1% and 3.5% in non‐poultry workers, respectively (MN assay; cutoff titer of 40).^43^ A serological study in Guangzhou, China, also showed that the prevalence of anti‐H9 antibodies was higher than for anti‐H5 antibodies (4.5% vs 0.2%) (HI and MN assays).^41^ The above‐mentioned findings indicate that the seroprevalence of antibodies against H9N2 virus was higher than that for other common AIVs. Two possible reasons for this finding exist. First, the Q226L mutation in the HA RBS of the H9N2 virus^14^ confers on it a greater ability to adapt to humans than the other AIVs possess.^44^ Second, the infection sources for people infected with H9N2 virus differ from those for H5N1. Specifically, people became infected with H9N2 through contact with healthy‐appearing poultry, whereas people became infected with H5N1 by contact with sick or dead poultry, because H5N1 is a highly pathogenic AIV.^45^ Generally, we found that people who lacked occupational exposure also had more opportunities to make contact with healthy‐appearing poultry than with sick or dead poultry. Hence, compared with other AIVs, H9N2 may pose a risk to a wider range of people, and it is more difficult to prevent infections with it.

Our cohort study did not reveal any differences in the risk of contracting H9N2 virus between the swine workers and the general population, which is consistent with a previous study's findings.^41^ Indeed, the poultry workers had a higher risk than the general population of contracting an H9N2 infection, similar to that reported previously.^40^, ^41^ The higher risk of infection with H9N2 virus among poultry workers suggests that increased exposure to poultry would heighten the risk of contracting this virus. As many people frequently visit live poultry markets, especially in southern China,^11^ it is important to enforce infection control measures in these places (eg, daily cleaning and disinfection, banning overnight poultry storage, and enforcing monthly rest days), because they represent one of the most important AIV reservoirs.^46^, ^47^

### 4.1 Limitations

There were several limitations in our study. First, it cannot be excluded that the antibodies against H9N2 virus detected by the HI assay were not confounded by antibodies against other H9 viruses or even antibodies against N2 among the participants who lived through the H2N2 pandemic. Second, in comparison with the general population, there were greater losses to follow‐up among the poultry workers and swine workers because most of these occupational populations in Beijing were migrant workers from other provinces, and this would have led to high turnover rates among them. Additionally, as the migrant workers came from other provinces where the circulation intensity of the H9N2 virus differed from that in Beijing or where live poultry may be allowed in the markets, this would have affected the evaluation of previous infections in the cross‐sectional surveys. Third, the incidence of H9N2 viral infections was determined by antibody seroconversion, but the incidence of symptomatic infections confirmed by virological assays was not investigated.

## 5 CONCLUSION

Although the overall level of infection with H9N2 virus was low in Beijing, China, the poultry workers were at higher risk of infecting H9N2 viral infections than the general population. Closer monitoring and strengthened protection measures for poultry workers are warranted.

## CONFLICT OF INTEREST

The authors report no conflict of interests.

## AUTHOR'S CONTRIBUTION

Wang QY and Yang P conceived the study; Yang P and Ma CN designed the study; Ma CN, Sun Y, Zhang L, Zhang Y, Wu SS, Duan W, and Zhang M performed the data collection; Cui SJ, Zhao JC, Zhang DT, and Pan Y performed the experiments; Ma CN drafted the study; Yang P and Wang QY revised the study. All the authors revised and approved the final version of the manuscript.
