# Supplementary material for: Avian influenza A (H9N2) virus infections among poultry workers, swine workers, and the general population in Beijing, China, 2013‐2016: A serological cohort study
Source: Influenza Other Respir Viruses. 2019 Mar 18;13(4):415–25. doi: 10.1111/irv.12641 (PMC6586185; doi:10.1111/irv.12641)
Supplement: Supplementary file 1 [file IRV-13-415-s001.docx]

Appendix

Table 1 Illustration of how single serum samples from the four cross-sectional surveys (from November 2013 to April 2016) were determined to be positive.

|  | Nov 2013 | |  | Apr 2014 | |  | Apr 2015 | |  | Apr 2016 | |
| --- | --- | --- | --- | --- | --- | --- | --- | --- | --- | --- | --- |
|  | HI titer | P/N |  | HI titer | P/N |  | HI titer | P/N |  | HI titer | P/N |
| Criteria | ≥80 | P |  | ≥80 | P |  | ≥80 | P |  | ≥80 | P |
| Hypothetical Examples | |  |  |  |  |  |  |  |  |  |  |
| Participant 1 | 640 | P |  | 320 | P |  | 160 | P |  | 80 | P |
| Participant 2 | 80 | P |  | 160 | P |  | 320 | P |  | 640 | P |
| Participant 3 | 80 | P |  | 160 | P |  | 80 | P |  | 40 | N |
| Participant 4 | 80 | P |  | 160 | P |  | 40 | N |  | 80 | P |
| Participant 5 | 40 | N |  | 160 | P |  | 80 | P |  | 40 | N |
| Participant 6 | 40 | N |  | 160 | P |  | 40 | N |  | 80 | P |
| Participant 7 | - | - |  | - | - |  | 80 | P |  | 80 | P |
| Participant 8 | - | - |  | 80 | P |  | - | - |  | 80 | P |
| Participant 9 | 80 | P |  | - | - |  | - | - |  | - | - |

Single serum samples each with a titer of 80 or more were considered as positive in four dependent surveys.

P denotes positive; N denotes negative.

“-” denotes that the participant did not participate in this year’s survey.

Table 2 Illustration of how seroconversion in the paired serum samples from our cohort study (from November 2013 to April 2016) was determined.

|  | HI titer | | | |  | Seroconversion（Y/N/E） | | |
| --- | --- | --- | --- | --- | --- | --- | --- | --- |
|  | Nov 2013 | Apr 2014 | Apr 2015 | Apr 2016 |  | 2013-2014 year | 2014-2015 year | 2015-2016 year |
| Hypothetical Examples | |  |  |  |  |  |  |  |
| Participant 1 | 10 | 20 | 40 | 320 |  | N | N | Y |
| Participant 2 | 320 | 160 | 80 | 40 |  | N | N | N |
| Participant 3 | 10 | 40 | 10 | 40 |  | Y | N | Y |
| Participant 4 | 10 | 40 | - | 40 |  | Y | E | E |
| Participant 5 | 10 | 40 | - | 160 |  | Y | E | E |
| Participant 6 | 10 | - | - | 160 |  | E | E | E |

Seroconversion of antibodies against H9N2 virus have a 4-fold or greater increase between the paired serum specimens with a titer ≥ 40 for the second specimen.

P denotes positive; N denotes negative; E denotes exclusive.

“-” denotes that the participant did not participate in this year’s survey.
